# Utility of ABI and API Versus CTA to Identify Surgically Significant Arterial Injury After Lower Extremity Trauma in a LMIC

**DOI:** 10.1002/wjs.12623

**Published:** 2025-05-19

**Authors:** Rubinette Robbertze, Megan Lubout, Daniel Nicholas Prince, Isabella Margaretha Joubert, Maeyane S. Moeng

**Affiliations:** ^1^ Division of Diagnostic Radiology Department of Radiation Sciences School of Clinical Medicine Faculty of Health Sciences University of the Witwatersrand Johannesburg South Africa; ^2^ Trauma Surgery Trauma Surgeon Mater Misericordiae University Hospital Dublin Ireland; ^3^ Division of General Surgery School of Clinical Medicine Faculty of Health Sciences University of the Witwatersrand Johannesburg South Africa; ^4^ Radiology Department Dunedin Hospital Dunedin New Zealand; ^5^ Department of Trauma Surgery, Division of General Surgery, School of Clinical Medicine, Faculty of Health Sciences University of the Witwatersrand Johannesburg South Africa; ^6^ Trauma Unit Charlotte Maxeke Johannesburg Academic Hospital Johannesburg South Africa; ^7^ Trauma Unit Milpark Netcare Hospital Johannesburg South Africa

**Keywords:** ankle‐brachial index, arterial injury, arterial pressure index, CT angiography, lower extremity trauma, utilization

## Abstract

**Background:**

South Africa faces a high burden of trauma‐related vascular injury. Prompt diagnosis and management are crucial to limit morbidity and mortality. Literature recommends a thorough vascular examination of at‐risk patients. Ankle brachial index (ABI) and arterial pressure index (API) are considered reliable screening tools for lower extremity vascular injury (LEVI) in the correct clinical scenario. Patients with an abnormal ABI/API warrant diagnostic imaging with computed tomography angiography (CTA). However, recent international literature demonstrates a trend toward potential CTA overuse in the work up for LEVI, when the internationally recommended vascular injury work‐up guidelines are not followed correctly.

**Aim:**

To assess the reliability of ABI/API in trauma patients with suspected LEVI as a screening tool to safely avoid unnecessary CTA.

**Methods:**

A retrospective cohort study of all lower extremity trauma patients with soft signs of LEVI who presented to Charlotte Maxeke Johannesburg Academic Hospital from February 1, 2018 to January 31, 2020 was undertaken. Sensitivity, specificity, NPV, and PPV were calculated for ABI/API versus CTA and ABI/API/CTA versus surgically significant arterial injury. A *p*‐value < 0.05 indicated statistical significance (confidence level = 95%).

**Results:**

Four hundred and thirty‐three CTAs were performed for suspected traumatic LEVI. Two hundred and eighty‐two were excluded due to missing data (precluding retrospective calculation of ABI/API) and 151 patients were included. To detect surgically significant injury, CTA had a 100% sensitivity, 97.2% specificity, 100% NPV, and 69.2% PPV; ABI and API had a 100% sensitivity, 83.8%–85.9% specificity, 100% NPV, and 28.1%–35.9% PPV, respectively. Neither ABI nor API missed surgically significant arterial injuries.

**Conclusion:**

This affirms the reliability of ABI/API as a screening tool to identify patients at risk of LEVI from penetrating trauma. Findings supported international data demonstrating CTA overuse in this subset of patients.

## Graphical abstract

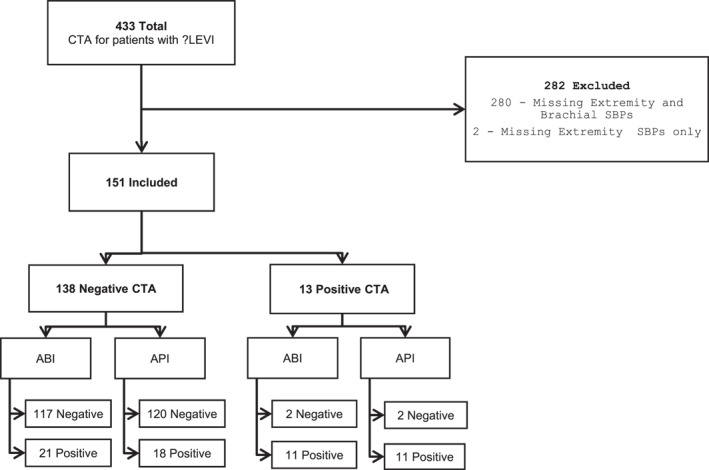

**Abbreviations**
ABI: ankle‐brachial index
API: arterial pressure index
AUC: area under the ROC curve
AVF: arteriovenous fistula
BP: blood pressure
CMJAH: Charlotte Maxeke Johannesburg Academic Hospital
CTA: computed tomography angiography
ED: emergency department
GSW: gunshot wound
HREC: Human Research Ethical Committee
LEVI: lower extremity vascular injury
LMIC: lower middle‐income country
MOI: mechanism of injury
MVC: motor‐vehicle collision
NPV: negative predictive value
PACS: picture archiving and communication system
PAD: peripheral arterial disease
PPV: positive predictive value
PVC: pedestrian‐vehicle collision
ROC: receiver operating curve
SBP: systolic blood pressure

## 1 Introduction

Injury and trauma‐related ailments form part of the quadruple burden of disease in South Africa, a lower middle‐income country (LMIC) [1]. A recent article from 2024 demonstrated that trauma accounted for the majority (36%) of emergency department (ED) visits to a district hospital in North–West province, South Africa, mirroring presentation nationally [2, 3, 4]. This trauma burden results in significant morbidity and mortality annually, resulting in overburdened and understaffed ED and healthcare facilities [4, 5, 6, 7].

Trauma‐related vascular injuries are a leading cause of morbidity and mortality globally [8]. Although there is limited data regarding the incidence of extremity vascular trauma in South Africa, these types of injuries are a known and common cause of ED presentation. Generally, lower extremity vascular injury (LEVI) is more prevalent (50%–60%) than upper limb vascular injury [9, 10].

To limit morbidity and mortality related to these injuries in a low‐middle income country (LMIC) with a high burden of trauma‐related injury, prompt diagnosis and timely management are crucial. International literature recommends that a thorough physical examination (extremity pulse status and assessment for soft and hard signs of vascular injury) should be performed on all at‐risk patients. Those with hard signs of vascular injury (summarized in Table 1) necessitate operative intervention. In those with soft signs, comprehensive physical examination should be followed by screening vascular tests, such as ankle‐brachial index (ABI) and arterial pressure index (API), to determine the need for further imaging and management [9, 10].

**TABLE 1:** Hard and soft signs of vascular injury.

Hard signs	Soft signs
External bleeding	History of arterial bleeding on scene or during transfer
Rapidly expanding hematoma	Injury in proximity to a major or named artery
Palpable thrill or audible bruit	Small nonpulsatile hematoma over a major or named artery
Classical signs of arterial occlusion (pulselessness, pallor, paresthesia, pain, and paralysis)	Neurological deficit in the distribution of a peripheral nerve adjacent to a major or named artery [9, 10]

ABI and API are the two most widely recognized screening vascular tests utilized in patients with potential traumatic LEVI [9, 10]. ABI is defined as the ratio of the systolic blood pressure (SBP) in the extremity distal to the level of injury to the SBP in the brachial artery of an uninjured upper extremity. API is defined as the ratio of the Doppler arterial pressure of the injured extremity distal to the level of injury to the Doppler arterial pressure in the uninjured extremity.

Internationally, an ABI/API threshold value of ≥ 0.9 has the highest combined specificity (> 90%) and negative predictive value (98%) when evaluating the likelihood of LEVI requiring intervention in patients with soft signs of vascular injury [9, 10, 11]. In the absence of other life‐threatening injuries, current international guidelines advise that patients with soft signs of vascular injury require diagnostic imaging only if the ABI or API is abnormal (< 0.9). In contrast, those with a normal ABI or API can be safely discharged for outpatient follow‐up. Mandatory outpatient follow‐up of these patients is recommended using both EAST and WTA guidelines, to identify potential initially undetected vascular injuries (1%–4%) or complications, most commonly soft tissue infections (5.5%) [9, 10].

However, there are recognized clinical scenarios where the applicability of API or ABI are limited, including hemodynamically unstable patients, hypothermia, obesity, and patients with preexisting peripheral arterial disease (PAD) [9, 10]. It is recommended that patients with hemodynamic instability be resuscitated before vascular examination is performed or relied upon. In obese patients, the literature recommends using an appropriately sized blood pressure (BP) cuff when performing vascular screening tests. In patients with known PAD, a higher threshold ABI or API value (≥ 1.0) has been suggested [9, 10]. Care should also be taken in patients with multilimb trauma.

If diagnostic vascular imaging is indicated, peripheral computed tomography angiography (CTA) has surpassed conventional digital subtraction angiography (DSA) as the international gold standard. Advantages of CTA over DSA include widespread availability, reduced costs, high interobserver agreement, intravenous only contrast injection negating the need for arterial puncture, and related complications [9, 10, 12].

However, recent international literature has demonstrated a trend toward overutilization of CTA in the workup of suspected LEVI, since it is increasingly used as the initial screening tool for identification of vascular injury prior to dedicated vascular examination and screening tools such as ABI/API [13, 14, 15]. This is contrary to international guidelines recommending the use of CTA as an adjunct to vascular screening tools. This trend may be associated with unnecessary costs incurred by the facility and patients, increased ED waiting times, increased radiation dose, contrast‐related complications, and increased workload and costs for the already overburdened radiology departments [4, 13, 14].

Furthermore, access to CT imaging in South Africa is limited, since there are only approximately 5 CT machines available per 1 million population compared to the USA, which has approximately 43 CT machines per million population [16]. Evidently, CT imaging is a scarce resource in this LMIC setting and strategies to optimize its utilization are necessary.

In this LMIC setting, where public healthcare systems are overburdened and understaffed, utilization of international vascular injury work‐up guidelines could safely eliminate unnecessary CTA imaging, thereby decreasing costs, emergency room patient overcrowding, and the workload of ED and radiology departments alike.

## 2 Aim of the Study

To assess the reliability of ABI and API in trauma patients with soft signs of LEVI as screening tools to safely avoid unnecessary CTA.

## 3 Method

### 3.1 Study Design

A retrospective cohort study of all lower extremity (groin to knee) trauma patients who presented with soft signs of vascular injury to Charlotte Maxeke Johannesburg Academic Hospital (CMJAH) trauma department from February 1, 2018 to January 31, 2020 was undertaken.

Ethics approval from the University of Witwatersrand Health Research Ethical Committee (HREC) (ref. no M220825) and institutional approval from the facility Chief Executive Officer were obtained.

Patients 18 years and older who had recorded vascular examination findings (bilateral lower limb and brachial artery BPs) and subsequently received lower extremity CTA were included. Those who presented with hemodynamic instability, hard signs of vascular injury, had injuries above the level of the common femoral artery or below the popliteal trifurcation, patients with bilateral lower extremity injury, and those who did not have recorded vascular examination findings (bilateral lower limb and/or brachial BPs) were excluded.

Data were retrospectively collected from the ED records, resuscitation forms, hospital files, surgical notes, and the institution's picture archiving and communication system (PACS). Data collected was anonymized and documented on a password‐protected Microsoft Excel spreadsheet for further analysis. The data collected included patient demographics; mechanism of injury (MOI); type of injury (i.e., gunshot wound [GSW] or motor vehicle collision [MVC]); brachial artery SBP; SBP of the injured and uninjured limbs; timing of CTA post trauma, CTA findings; and timing of surgery post injury, details of vascular surgical intervention if any.

The ABI and API were calculated retrospectively by the authors, using the admission brachial artery and bilateral lower limb SBPs as recorded in the patient resuscitation forms. It is unclear from patient records who measured the SBPs; however, in the study Center, these examinations are generally performed by a trauma nurse under the supervision of a medical doctor. For this study, an ABI/API threshold value equal to 0.9 or higher was considered normal, corresponding to current international standards.

For the purposes of this study, “surgically significant arterial injury” was defined as arterial injury, which required surgical intervention.

### 3.2 Statistical Analysis

Data were analyzed using IBM SPSS version 28. Descriptive statistics were used to demonstrate patients' demographic profiles and clinical characteristics. Categorical variables were reported using frequencies and percentages, and continuous scale data were reported in means with standard deviation or median with interquartile range. The sensitivity, specificity, NPV, and PPV of ABI and API to detect arterial injury were computed in crosstabulations in which CTA was treated as the gold standard. The sensitivity, specificity, NPV, and PPV of ABI, API, and CTA to detect significant injury were computed in crosstabulations in which injuries requiring surgical intervention was treated as the gold standard were also calculated. The receiver operating curves (ROC) and area under the curve (AUC) for ABI, API, and CTA were calculated. The Cohen's kappa statistic was used to measure the level of agreement between the alternative methods. Statistical significance testing was set at the 95% confidence level, and therefore, *p* < 0.05 indicated statistical significance.

## 4 Results

**FIGURE 1:**
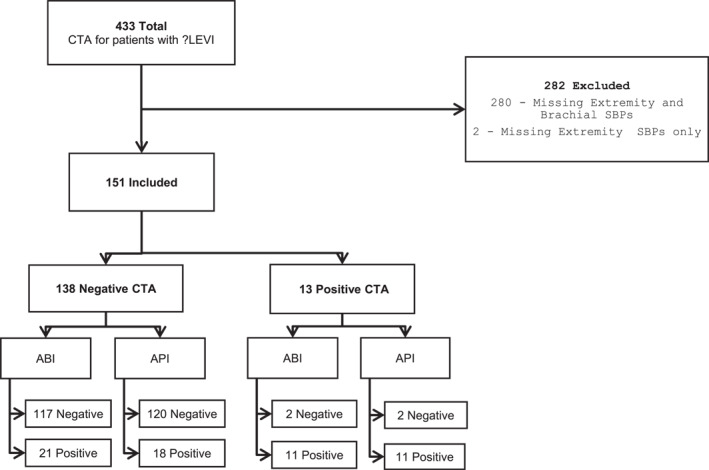
Flow diagram demonstrating total, included, and excluded CTAs with corresponding ABI/API results where applicable.

There was a total of 433 peripheral CTA performed for suspected traumatic LEVI at our facility during the study period. Of these, 282 patients (65%) had to be excluded as they were referred for CTA without documentation of brachial and/or bilateral lower extremity BP, which precluded retrospective calculation of ABI and API (see Figure 1). Similar to the included population where 91.4% had a negative CTA, a substantial majority (91.8%) of the excluded patients had a negative CTA where no vascular injury was demonstrated.

Of the 151 included patients (34.9%), most were men (94.7%, *n* = 143) and penetrating trauma (93.4%, *n* = 141) accounted for the most frequent MOI, of which gunshot wound injuries (86.8%) were the most prevalent (see Table 2). The mean age of presentation was 31.8 years, with an interquartile range of 18.8–44.8 years.

**TABLE 2:** Participant characteristics.

	Frequency	Percentage
Sex		
Female	8	5.3
Male	143	94.7
Total	151	100
Age		
18–24	35	23.2
25–44	100	66.2
45–64	13	8.6
≥ 65	2	1.3
Unknown	1	0.7
Total	151	100
Mechanism of injury		
Blunt	10	6.6
Penetrating	141	93.4
Total	151	100
Type of injury		
MVC	1	0.7
PVC	4	2.6
Fall	6	4.0
GSW	131	86.8
Stab	9	6.0
Total	151	100

Abbreviations: GSW, gunshot wound; MVC, motor‐vehicle collision; PVC, pedestrian‐vehicle collision.

### 4.1 CTA Versus Surgically Significant Injury

Most of the included CTA studies (*n* = 138, 91%) were normal with no arterial injury. There were 13 (8.6%) positive CTA studies, which demonstrated arterial injury (see Figure 1). Of these, nine patients (69.2%) required surgical intervention and were considered surgically significant, whereas four patients (31.8%) did not require surgical intervention per trauma and vascular surgical decision and were discharged home (summarized in Table 3).

**TABLE 3:** Summary of injuries found on positive CTA with description of surgical intervention.

		CTA findings	Surgical management
Required surgery	1	SFA and profunda femoris injury with thrombosis and long segment SFA cutoff	Fasciotomy, SFA exploration, and PTFE graft
	2	Distal popliteal artery cutoff	Popliteal artery exploration, distal popliteal repair with PTFE graft, and fasciotomy
	3	Distal SFA injury and thrombosis	SFA exploration and primary repair
	4	SFA injury and pseudoaneurysm	SFA exploration and PTFE graft
	5	SFA cutoff	SFA repair with RSVG
	6	Tibialis posterior artery cutoff	Fasciotomy for compartment syndrome, no vascular intervention
	7	SFA cutoff	SFA primary repair
	8	Mid SFA cutoff	SFA exploration and primary repair
	9	Mid SFA cutoff	SFA exploration and RSVG
Did not require surgery	1	Superficial femoral vein early venous filling	None
	2	Tibialis anterior artery cutoff	None
	3	Tibialis anterior and tibialis posterior cutoff	None
	4	Popliteal artery early venous filling	None

Abbreviations: PTFE, polytetrafluoroethylene; RSVG, reverse saphenous vein graft; SFA, superficial femoral artery.

**FIGURE 2:**
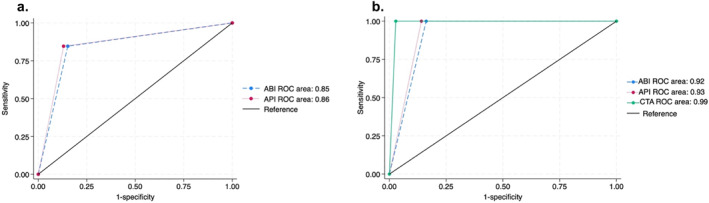
ROC for predicting traumatic lower extremity arterial injury using various tests. (a) ROC for predicting traumatic lower extremity arterial injury by ABI and API compared to CTA. Blue dashed line represents ABI with an AUC of 0.85. Red dotted line represents API with an AUC of 0.86. (b) ROC for predicting surgically significant arterial injury after lower extremity trauma by ABI, API, and CTA compared to injuries found and repaired at operation. Blue dashed line represents ABI with an AUC of 0.92. Red dotted line represents API with an AUC of 0.93. Green solid line represents CTA with AUC of 0.99.

For detection of arterial injury requiring surgical intervention, CTA had a sensitivity of 100%, specificity of 97.2%, NPV of 100%, PPV of 69.2% (summarized in Table 4), and an AUC of 0.99 (plotted in Figure 2b). In this study, CTA did not miss any surgically significant arterial injuries.

**TABLE 4:** Sensitivity, specificity, NPV, PPV, level of agreement, and area under the ROC curve of various tests to detect surgically significant arterial injury after lower extremity trauma compared to CTA and injuries found at operation.

	Sensitivity (95% CI)	Specificity (95% CI)	NPV (95% CI)	PPV (95% CI)	Level of agreement		Area under the ROC	
					Kappa (95% CI)	*p*‐value	Area (95% CI)	Asymptotic significance
Compared to CTA								
ABI	84.6% (54.6%–98.1%)	84.8% (77.7%–90.3%)	98.3% (94.1%–99.8%)	34.4% (18.6%–53.2%)	0.41 (0.35–0.49)	< 0.001	0.85 (0.73–0.97)	< 0.001
API	84.6% (54.6%–98.1%)	87% (80.2%–92.1%)	98.4% (94.2%–99.8%)	37.9% (20.7%–57.7%)	0.46 (0.39–0.53)	< 0.001	0.86 (0.74–0.98)	< 0.001
Compared to injuries found at operation								
ABI	100% (66.4%–100%)	83.8% (76.7%–89.4%)	100% (96.9%–100%)	28.1% (13.7%–46.7%)	0.38 (0.32–0.45)	< 0.001	0.92 (0.87–0.97)	< 0.001
API	100% (66.4%–100%)	85.9% (79.1%–91.2%)	100% (97.0%–100%)	31% (15.3%–50.8%)	0.42 (0.26–0.49)	< 0.001	0.93 (0.89–0.97)	< 0.001
CTA	100% (66.4%–100%)	97.2% (92.9%–99.2%)	100% (97.4%–100%)	69.2% (38.6%–90.9%)	0.80 (0.73–0.86)	< 0.001	0.99 (0.97–1.00)	< 0.001

Abbreviations: CI, confidence interval; kappa, Cohen's kappa value; NPV, negative predictive value; PPV, positive predictive value.

### 4.2 ABI Versus CTA

Of the 138 patients with negative CTA results, 117 had a normal ABI (true negative) and 21 had an abnormal ABI (false positive). Of the 13 patients with positive CTA, 11 had an abnormal ABI (true positive).

There were two patients with a false negative ABI. The first patient had early venous filling on CTA; however upon imaging and clinical review, an arteriovenous fistula (AVF) was ruled out and the patient was discharged home. The second patient had an isolated tibialis anterior artery cutoff and did not require surgical intervention. Therefore, screening ABI identified all patients requiring surgical intervention (surgically significant injury).

When compared to CTA, ABI had a specificity of 84.8%, sensitivity of 84.6%, NPV of 98.3%, PPV of 34.4% (refer to Table 4), and AUC of 0.85 in the detection of post‐traumatic LEVI (refer to Figure 2a).

### 4.3 API Versus CTA

Of the 138 patients with negative CTA results, 120 had a normal ABI (true negative) and 18 had an abnormal ABI (false positive). Of the 13 patients with positive CTA, 11 had an abnormal ABI (true positive) and the same 2 patients detailed under ABI versus CTA were found to have a false negative API, which were not considered surgically significant.

When compared to CTA, API had a specificity of 87%, sensitivity of 84.6%, NPV of 98.4%, PPV of 37.9% (refer to Table 4), and AUC of 0.86 in the detection of post traumatic LEVI (refer to Figure 2a).

### 4.4 ABI/API Versus Surgically Significant Injury

ABI had a sensitivity of 100%, specificity of 83.8%, NPV of 100%, PPV 28.1%, and AUC of 0.92 to detect surgically significant post‐traumatic LEVI, whereas API had a sensitivity of 100%, specificity of 85.9%, NPV of 100%, PPV of 31% (refer to Table 4), and AUC of 0.93 (refer Figure 2b for detail).

### 4.5 Indicated Versus Nonindicated CTA According to International Guidelines

Of the 151 patients who underwent peripheral CTA for soft signs of LEVI after lower extremity trauma, only 32 (21.2%) and 29 (19.2%) had a positive ABI and API, respectively, thus warranting further imaging according to international guidelines. Conversely, 119 (78.8%) and 122 (80.8%) had a negative ABI and API, respectively, which should have negated the need for further imaging according to international guidelines.

## 5 Discussion

Our study population size (*n* = 151) is comparable to similar international studies (Kelly et al., *n* = 157) but is presently the largest sample size locally, which specifically assesses the reliability of ABI and API to detect surgically significant LEVI [17]. Akin to other international and local studies, most of our study patients were male (94.7%), with penetrating trauma, the most common etiology for lower extremity injury [9, 10].

Although one of our study objectives were to evaluate the reliability of ABI and API in both penetrating and blunt trauma, only 10 patients (6.6%) sustained blunt lower limb trauma with none having vascular injuries requiring revascularization. As such, we were unable to accurately assess the reliability of these screening tests in blunt lower extremity trauma.

This study confirms excellent sensitivity (100%), specificity (100%), and NPV (100%) of peripheral CTA for detecting surgically significant arterial injury post lower extremity trauma, in keeping with international data [9, 10]. Furthermore, it demonstrated a low PPV (69.2%) for CTA in detection of arterial injury requiring vascular intervention, which is consistent with results from a recent retrospective review at a Level 1 trauma center by Kelly et al. [17].

When applying the standard international threshold value of 0.9, ABI and API demonstrated high specificity (ABI 83.8% and API 85.9%) and excellent sensitivity (100%) and NPV (100%) in predicting arterial injury requiring surgical intervention. In this cohort, neither ABI nor API missed any surgically significant arterial injuries requiring intervention. These findings support the use of both ABI and API as screening tools to effectively identify patients requiring further imaging to confirm or rule out surgically significant injury, and those who do not require further imaging who can therefore be discharged home for outpatient follow‐up, consistent with international data [9, 10].

Approximately two‐thirds of the 433 patients referred for CTA for suspected LEVI over the 2‐year study period had to be excluded due to a lack of documentation of vascular examination findings. Most excluded patients (91.8%) had a negative CTA. This begs the question whether most of these CTA's could safely have been avoided.

Based on the results obtained from patients who met inclusion criteria (*n* = 151), by doing a simple physical vascular examination with API or ABI and applying internationally recognized cutoff values, 79.5% of these studies were unnecessary. Considering that each peripheral CTA performed at our institution currently costs approximately R4700 and takes at least 15 min to perform, approximately R564,000 of (± USD 31,996) unnecessary expenditure and at least 30 h of imaging time could have been saved by utilizing recognized international guidelines. This represents a significant potential saving for an institution in a low‐middle income country where resources are already very limited. Our results additionally support recent international data, which has demonstrated an overutilization of peripheral CTA after lower extremity trauma [13, 17].

## 6 Limitations

As this study is a retrospective review, it is inherently limited by the quality and accuracy of recorded data, including incompletely documented vascular examination findings. Given the absence of recorded vascular examination findings in up to two‐thirds of the total patients who received CTA for suspected LEVI, many patients had to be excluded from the sample, which may impact the accuracy of the results. This could be overcome by designing a prospective study with clearly defined data fields for collection and thus eliminating this source of bias.

Due to the low number of patients with blunt LEVI present in this cohort of patients, no inferences could be made about the reliability of ABI/API to detect significant vascular injuries in this population.

Care should be taken in those recognized clinical scenarios where the applicability of API/ABI is limited (hemodynamically instability, hypothermia, obesity, PAD, and bilateral lower extremity injury) [9, 10]. The mean age of presentation in this cohort was 31.8 years (interquartile range of 18.8–44.8 years), as such, PAD was not prevalent and the reliability of ABI/API in this scenario could not be assessed.

However, despite the identified limitations, we believe that the congruence between the results from this study and similar international studies affirms the utility and reliability of physical examination and screening vascular examination tests in the workup of suspected lower extremity trauma.

## 7 Conclusions

**FIGURE 3:**
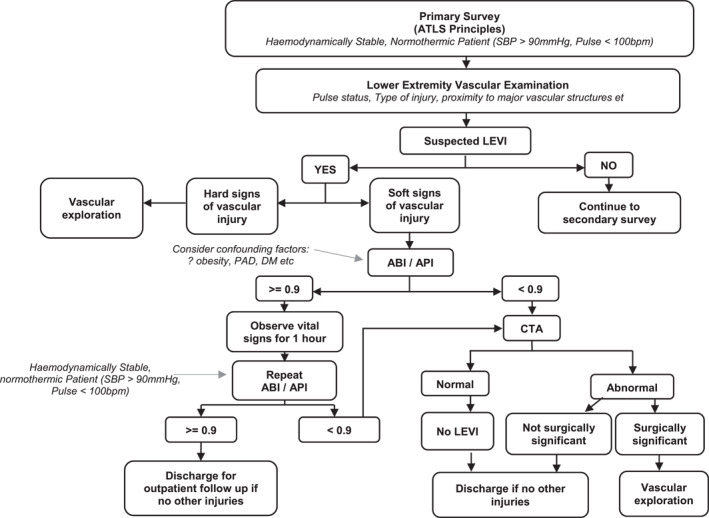
Flow diagram demonstrating proposed utilization of ABI/API in patients with suspected traumatic lower extremity vascular injury.

Our study affirms the reliability of ABI and API as a screening vascular examination tool in South African patients presenting with soft signs of vascular injury after unilateral lower extremity penetrating trauma. It further supports international data demonstrating CTA overuse in this subset of trauma patients. If applied correctly, we suggest ABI and API may be useful screening tests to limit unnecessary peripheral CTA after lower extremity trauma (refer to Figure 3 for the proposed protocol).

## 8 Future Perspectives

This study demonstrates the need for clear emergency department trauma protocols and selective referral for peripheral CTA after trauma.

Unfortunately, there is an absence of clear local or international guidelines regarding the timing of outpatient follow‐up in patients discharged from ED after lower extremity trauma to screen for missed vascular injury. We suggest that this is an important future avenue of study.

## Author Contributions

**Rubinette Robbertze:** conceptualization, investigation, writing – original draft, methodology, validation, visualization, writing – review and editing, formal analysis, data curation. **Megan Lubout:** conceptualization, methodology, validation, writing – review and editing, project administration, supervision, investigation, formal analysis. **Daniel Nicholas Prince:** investigation, methodology, validation, writing – review and editing, supervision, formal analysis. **Isabella Margaretha Joubert:** writing – review and editing, supervision, formal analysis. **Maeyane S. Moeng:** methodology, validation, writing – review and editing, project administration, supervision, formal analysis.

## Ethics Statement

Ethics approval was obtained from the University of Witwatersrand Health Research Ethical Committee (HREC) (ref. no M220825).

## Conflicts of Interest

The authors declare no conflicts of interest.

## Disclaimer

The views and opinions expressed in this article are those of the authors and do not necessarily reflect the official policy or position of any affiliated agency of the authors.

## Data Availability

Raw data were captured on a password‐protected Microsoft Excel spreadsheet. Derived data supporting the findings of this study are available from the corresponding author, Dr Rubinette Robbertze, on request.
